# Blunt Trauma-Induced Inferior Vena Cava Thrombosis and Renal Artery Injury With Abdominal Compartment Syndrome and Multi-organ Failure

**DOI:** 10.7759/cureus.109480

**Published:** 2026-05-23

**Authors:** Rohit Chauhan, Madhur Uniyal, Vasishta Nadella

**Affiliations:** 1 Trauma Surgery and Critical Care, All India Institute of Medical Sciences, Rishikesh, Rishikesh, IND

**Keywords:** abdominal blunt trauma, abdominal compartment syndrome (acs), acute kidney injury, inferior vena cava (ivc) thrombosis, multiple organ dysfunction syndrome (mods), renal vascular injury

## Abstract

Inferior vena cava (IVC) thrombosis is a rare but serious complication. We report a case of delayed presentation of abdominal compartment syndrome (ACS) secondary to IVC thrombosis, which contributed to progressive multi-organ dysfunction syndrome. Increased intra-abdominal pressure further resulted in venous stasis and thrombus formation, exacerbating systemic deterioration. Despite supportive care and attempted interventions, the patient developed refractory organ failure and expired. This case underscores the critical importance of early diagnosis and timely management of ACS to prevent vascular complications and fatal outcomes.

## Introduction

Inferior vena cava (IVC) thrombosis, renal artery injury, and abdominal compartment syndrome (ACS) represent a triad of interrelated, life-threatening pathologies that often converge in the setting of severe abdominal trauma or complex postoperative complications. Individually, each condition poses a serious threat to survival; in combination, they create a devastating cascade of hemodynamic and metabolic derangements requiring urgent, multidisciplinary intervention. Given the high morbidity and mortality associated with this triad, particularly in undiagnosed or delayed cases, early recognition is vital in patients with major abdominal or retroperitoneal trauma [1,2]. Aggressive, coordinated management includes continuous intra-abdominal pressure (IAP) monitoring, urgent imaging to delineate vascular involvement, cautious anticoagulation when bleeding risk allows, and timely decompressive laparotomy for refractory ACS to interrupt the cycle of organ failure and optimize outcomes [1]. This case report aims to elucidate the clinical course, management, and fatal progression of a patient with simultaneous IVC thrombosis and renal injury, which progressed to ACS.

## Case presentation

A 40-year-old male patient presented to our trauma emergency department approximately 20 hours after a road traffic accident. He was an unrestrained and helmetless pillion rider on a two-wheeler that collided head-on with a four-wheeler. He was ejected from the vehicle on impact and sustained a secondary collision with the four-wheeler. The patient was initially taken to a nearby hospital, where he received preliminary treatment with minimal resuscitation and was referred to a higher center. He arrived at our institute by ambulance approximately 20 hours after the accident.

At presentation, he was obtunded with a Glasgow Coma Scale score of E2V4M5 and no localizing signs. He was managed according to Advanced Trauma Life Support (ATLS) protocol. The right chest compression test was positive, with bilateral equal air entry and SpO₂ of 98% on room air. The extended Focused Assessment with Sonography in Trauma (eFAST) showed fluid in all four abdominal quadrants, with maximal collection in the pelvis.

He had a resting heart rate of 130 beats per minute and a noninvasive arterial blood pressure of 100/68 mm Hg while on inotropic support (inj. noradrenaline infusion at 65 mcg/min). His abdomen was tense and distended with generalized tenderness, and he had right-sided chest tenderness. No major external injuries were noted apart from minor abrasions.

Arterial blood gas analysis (Table 1) showed severe acidosis (pH 7.107, lactate 14.4, base deficit -12.4) and hyperkalemia (6.7 mmol/L). He was provisionally diagnosed with blunt trauma to the chest and abdomen with FAST-positive status and grade IV hemorrhagic shock (inotropic support confounded vital signs as markers of shock severity; lactate and base deficit were used to grade hemorrhagic shock severity, though the elevated lactate later appeared likely due to early-onset sepsis).

**Table 1:** Blood parameters at presentation ^*^ The hemoglobin disparity was due to the blood picture being ordered after the blood product transfusion. The arterial blood analyzer used was the Radiometer ABL800 FLEX model (Radiometer Medical ApS, Copenhagen, Denmark). ABG, arterial blood gas; Hb, hemoglobin; HCO₃⁻, bicarbonate; INR, international normalized ratio; K⁺, potassium; Na⁺, sodium; SGOT, serum glutamic-oxaloacetic transaminase; SGPT, serum glutamic-pyruvic transaminase

Parameter	Value	Reference range
ABG parameters		
pH	7.107	7.350-7.450
pCO₂	41.1 mm Hg	35.0-45.0 mm Hg
pO₂	87.9 mm Hg	83.0-108 mm Hg
HCO₃⁻	15.2 mEq/L	22-26 mEq/L
Hb	7.6 gm/dL	12.0-16.0 gm/dL
Lactate	14.4 mg/dL	0.5-1.5 mg/dL
Standard base deficit	12.4 mmol/L	0 ± 2 mmol/L
Na⁺	138 mEq/L	135-145 mEq/L
K⁺	6.7 mEq/L	3.5-4.5 mEq/L
Blood profile		
Hb	10.3^*^ gm/dL	12.0-16.0 gm/dL
Packed cell volume/hematocrit	30.80%	40-54%
Total leukocyte count	23,430 /µL	4,000-10,000 /µL
Platelet count	101,000 /µL	100,000-300,000 /µL
Renal parameters		
Urea	82 mg/dL	17-43 mg/dL
Creatinine	3.95 mg/dL	0.55-1.02 mg/dL
Na⁺	138 mmol/L	136-146 mmol/L
K⁺	6 mmol/L	3.5-5.1 mmol/L
Hepatic parameters		
Total bilirubin	4.28 mg/dL	0.3-1.2 mg/dL
Direct bilirubin	1.18 mg/dL	0-0.2 mg/dL
SGPT	1870 U/L	0-35 U/L
SGOT	4790 U/L	0-35 U/L
Gamma-glutamyl transferase	80 U/L	0-38 U/L
Alkaline phosphatase	97 U/L	30-120 U/L
Serum total protein	4.5 gm/dL	6.6-8.3 gm/dL
Serum albumin	2.8 gm/dL	3.5-5.2 gm/dL
Albumin/globulin ratio	1.6	1.1-2.1
Prothrombin time	15.5 seconds	9.8-12.1 seconds
INR	1.14	N/A

As per institutional guidelines, the blood bank was notified, and the Massive Transfusion Protocol was initiated. Balanced resuscitation with blood components in a 1:1:1 ratio was performed, with a total of four units each. Despite initial resuscitation, his hemodynamic parameters did not improve, and a repeat ABG showed persistent acidosis. He was then shifted to the radiodiagnosis unit for contrast-enhanced CT as per institutional trauma protocol.

**Figure 1:**
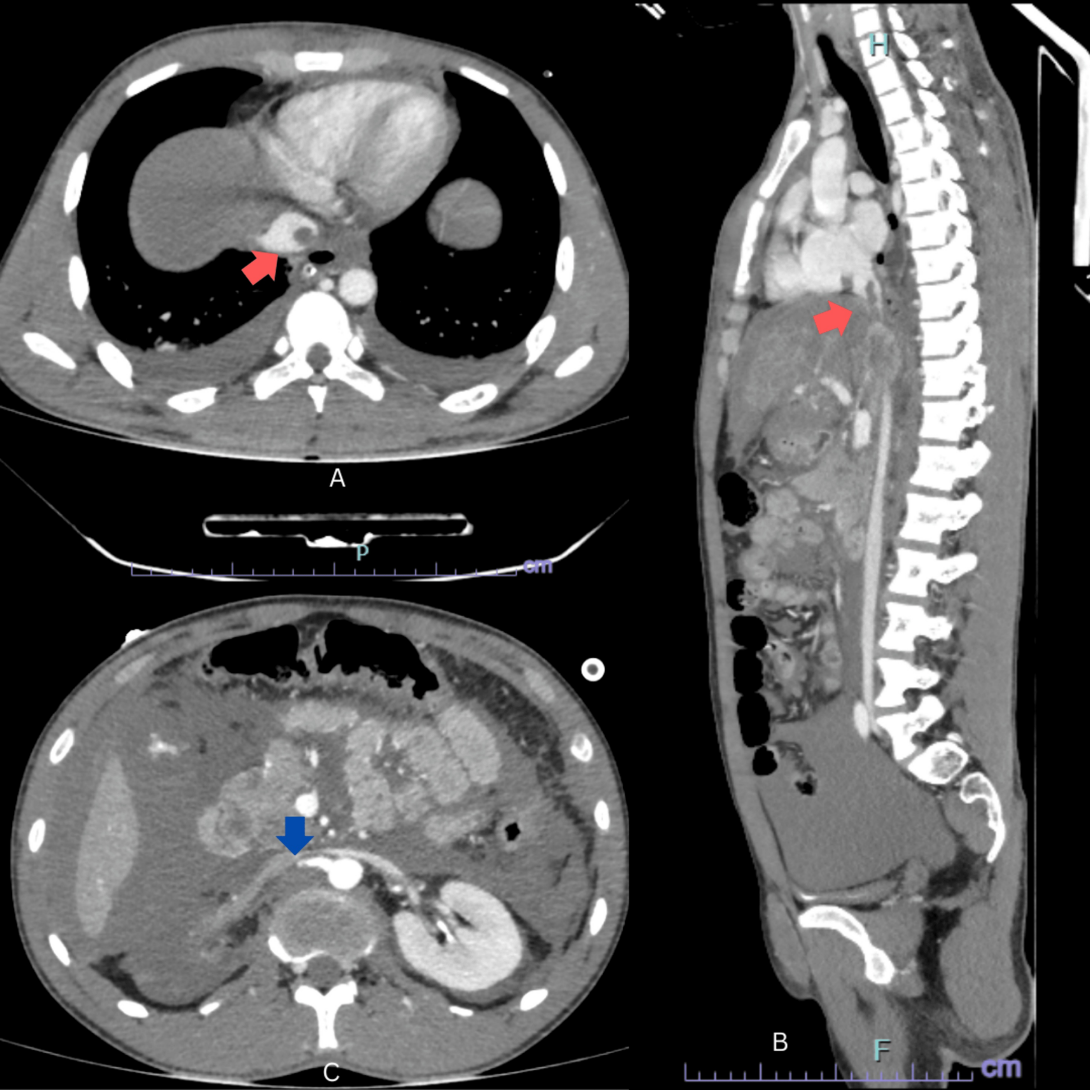
(A) Intraluminal thrombus in the suprahepatic portion of the IVC (red arrow). (B) Same thrombus in sagittal view (red arrow). (C) Abrupt cutoff of right renal artery opacification with non-enhancement of the right kidney, likely renal artery thrombosis (blue arrow). IVC, inferior vena cava

**Figure 2:**
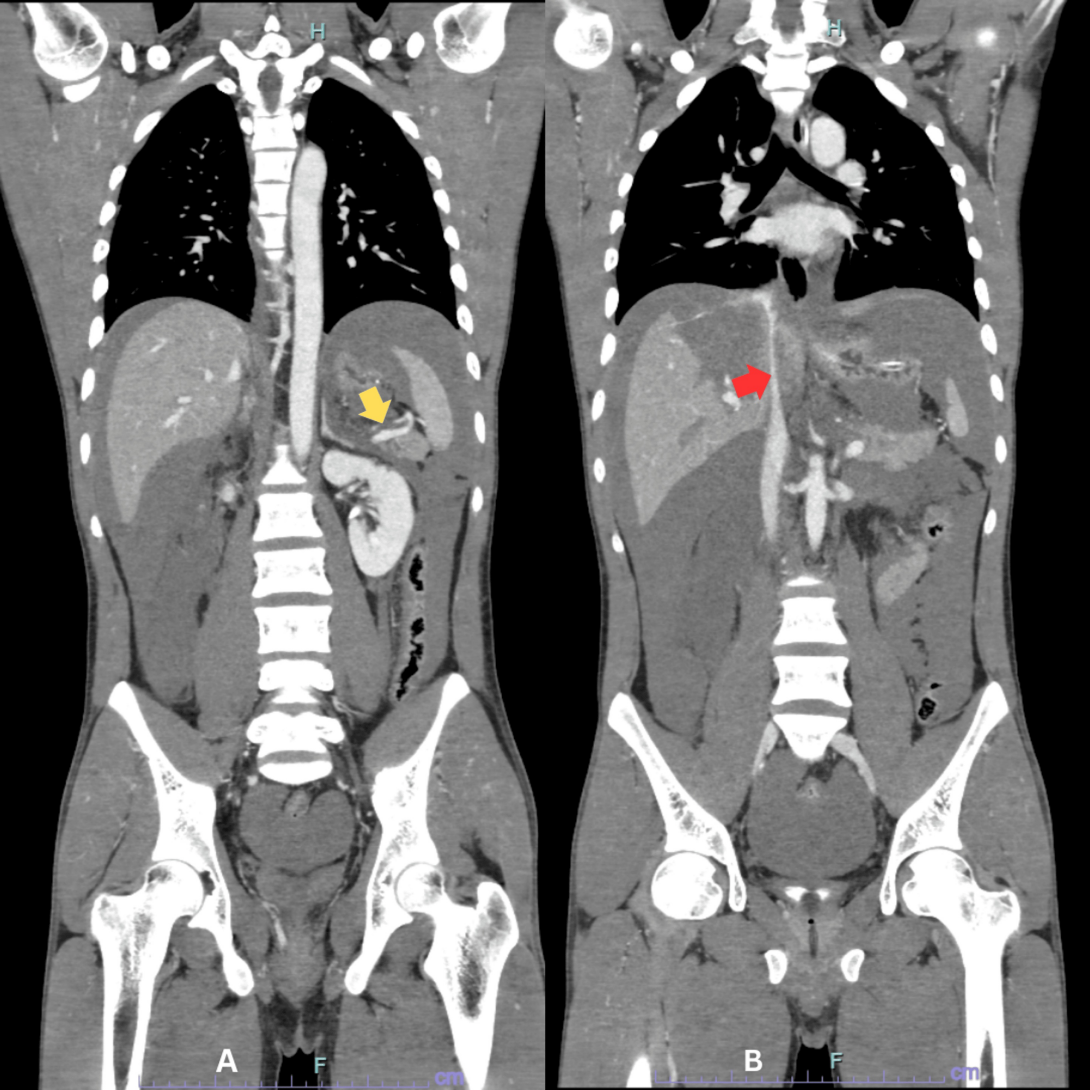
(A) Splenic vein thrombus (yellow arrow). (B) Same IVC thrombus (red arrow) with left hepatic vein thrombosis in coronal view. IVC, inferior vena cava

Contrast-enhanced CT showed a thrombus in the intrahepatic IVC extending to the suprahepatic portion, along with an American Association for the Surgery of Trauma (AAST) grade V liver injury (Figure 1, Figure 2) and right renal artery injury with AAST grade V renal injury (Figure 1). Other CT findings included AAST grade II/III pancreatic injury, left hepatic vein thrombus, and moderate hemoperitoneum. Noninvasive abdominal pressure (i.e., bladder pressure) measured via a water column manometer was 30 cm H₂O, exceeding the cutoff for ACS. The patient was therefore taken for emergency decompressive laparotomy and laparostomy as per the damage control surgery protocol. A total of 4 L of hemoperitoneum was evacuated, and an abdominal drain was placed in the pelvis. A lesser sac hematoma suggested retroperitoneal bleeding, and no bilious or feculent staining was observed. He was then immediately shifted to the ICU in an intubated state.

On Days 1 and 2, he underwent multiple hemodialysis sessions for refractory hyperkalemia (persistently >6.6 mmol/L). His oliguric state quickly progressed to anuria, and metabolic acidosis (pH 6.99-7.03) remained refractory. Despite decompression, acute kidney injury (AKI) progressed, and by the time of the fourth dialysis session, he was already on dual inotropic support (noradrenaline 1.4 mcg/kg/hr and vasopressin 0.03 U/min) with a mean arterial pressure of 60 mm Hg. Dialysis was halted as further hemodynamic instability ensued. No additional attempts at organ support could be made via continuous renal replacement therapy or extracorporeal membrane oxygenation. He expired on Day 5 due to septic shock with multiorgan failure, including liver failure and renal failure with refractory hyperkalemia.

## Discussion

Traumatic IVC thrombosis represents less than 1% of all blunt abdominal trauma cases [1,2]. Traumatic IVC thrombosis combined with renal artery injury represents a rare, complex, and life-threatening condition with significant diagnostic and therapeutic challenges. Blunt abdominal trauma can cause direct vascular injury, endothelial disruption, and compression from retroperitoneal or hepatic hematomas, all of which contribute to thrombus formation within the IVC. Concurrent renal injury, such as parenchymal, vascular, or both, as in our case, exacerbates the clinical situation by impairing renal venous outflow and renal function, often accelerating AKI and multi-organ failure, leading to rapid mortality if not promptly managed. Renal injury accompanying IVC thrombosis arises from direct trauma to renal vessels or parenchyma and impaired venous drainage, leading to increased venous pressure, ischemia, and renal dysfunction [1,3,4].

In our case, renal injury was further complicated by blocked arterial inflow. Although the renal vein was enhanced on initial CT slices, as time progressed, the IVC thrombosis could have hypothetically extended caudally to involve the renal segment of the IVC, causing obstruction of outflow from both kidneys. The presence of all these injuries can rapidly elevate IAP, and if not promptly diagnosed, can lead to ACS (defined as IAP >20 mm Hg with new organ dysfunction) [5], as occurred in our patient who experienced delayed definitive management by more than 24 hours. Early diagnosis primarily depends on high clinical suspicion in trauma patients with abdominal or flank injuries and signs suggestive of venous obstruction or renal impairment. Delayed diagnosis is common due to the rarity and nonspecific presentation, which adversely affects prognosis.

Prompt recognition and intervention are critical, as delays in decompressive laparotomy are associated with an increased risk of multi-organ failure and mortality [2,3,6]. Our case reaffirms the need for a high index of suspicion in trauma patients with escalating abdominal distension and persistent shock despite adequate resuscitation efforts. Routine measurement of IAP, typically via bladder pressure, is advocated in high-risk scenarios.

While this case shares features with existing reports on ACS after blunt trauma, it also illustrates the evolving challenges presented by modern resuscitative practices and damage-control surgery. Clinicians must be proactive in both prevention and early management.

Limitations of the current case include the inherent difficulty in distinguishing ACS-related organ dysfunction from other post-traumatic etiologies and the need for prospective, multicenter data to refine risk stratification and treatment algorithms for this population. Treatment decisions in traumatic IVC thrombosis with renal injury must balance the risk of hemorrhage from coexisting trauma and increasing abdominal pressure with the risks of thrombus propagation and embolization.

## Conclusions

In blunt abdominal trauma, especially with delayed presentation, there must be a high suspicion of intra-abdominal hypertension. In cases of liver injury, it is imperative to rule out IVC thrombosis, which, if missed, may be fatal, as in our case. The outcome reflects the catastrophic interplay between vascular injury, metabolic derangement, and multi-organ dysfunction. High suspicion, early imaging, periodic noninvasive abdominal (via bladder) pressure monitoring, and prompt decompression are crucial for improving survival in similar presentations. This case emphasizes the need for trauma systems that enable early referral and rapid multidisciplinary intervention.
